# Molecular basis for catalysis and substrate-mediated cellular stabilization of human tryptophan 2,3-dioxygenase

**DOI:** 10.1038/srep35169

**Published:** 2016-10-20

**Authors:** Ariel Lewis-Ballester, Farhad Forouhar, Sung-Mi Kim, Scott Lew, YongQiang Wang, Shay Karkashon, Jayaraman Seetharaman, Dipanwita Batabyal, Bing-Yu Chiang, Munif Hussain, Maria Almira Correia, Syun-Ru Yeh, Liang Tong

**Affiliations:** 1 Department of Physiology and Biophysics Albert Einstein College of Medicine Bronx, NY 10461, USA; 2 Department of Biological Sciences Northeast Structural Genomics Consortium Columbia University New York, NY 10027, USA; 3 Departments of Cellular and Molecular Pharmacology, Pharmaceutical Chemistry, and Bioengineering and Therapeutic Sciences, The Liver Center, University of California at San Francisco San Francisco, CA 94158, USA

## Abstract

Tryptophan 2,3-dioxygenase (TDO) and indoleamine 2,3-dioxygenase (IDO) play a central role in tryptophan metabolism and are involved in many cellular and disease processes. Here we report the crystal structure of human TDO (hTDO) in a ternary complex with the substrates *L*-Trp and O_2_ and in a binary complex with the product *N*-formylkynurenine (NFK), defining for the first time the binding modes of both substrates and the product of this enzyme. The structure indicates that the dioxygenation reaction is initiated by a direct attack of O_2_ on the C_2_ atom of the *L*-Trp indole ring. The structure also reveals an exo binding site for *L*-Trp, located ~42 Å from the active site and formed by residues conserved among tryptophan-auxotrophic TDOs. Biochemical and cellular studies indicate that Trp binding at this exo site does not affect enzyme catalysis but instead it retards the degradation of hTDO through the ubiquitin-dependent proteasomal pathway. This exo site may therefore provide a novel *L-*Trp-mediated regulation mechanism for cellular degradation of hTDO, which may have important implications in human diseases.

TDO and IDO are heme proteins that catalyze the oxidative cleavage of *L*-Trp (Supplementary Fig. 1), the first and rate-limiting step of the kynurenine pathway for *L*-Trp catabolism^1^,^2^,^3^,^4^,^5^,^6^,^7^. Trp is the least abundant essential amino acid. The majority of dietary Trp (~95%) is metabolized in the liver through this pathway to produce NAD^+^, while a small amount (~1%) is utilized to synthesize serotonin and melatonin through the serotonin pathway. TDO hence plays an important role in controlling the relative Trp flux along the two pathways. Its up-regulation can lead to over-production of neuroactive metabolites as well as serotonin deficiency. In *Drosophila*, TDO is known as *vermilion*, responsible for the bright-red eye color, due to its involvement in ommochrome biosynthesis^8^. TDO and IDO have been linked to a variety of human diseases including Alzheimer’s, Huntington’s, depression-associated anxiety, schizophrenia, and autism^9^,^10^,^11^,^12^,^13^,^14^,^15^,^16^. Recently, it was discovered that, in addition to the liver, TDO is highly expressed in certain cancer cells, where it plays a critical role in suppressing anti-tumor immunity via activating the aryl hydrocarbon receptor^17^,^18^. These discoveries have triggered a great deal of new interest in targeting TDO and IDO for drug discovery^19^,^20^,^21^,^22^,^23^,^24^.

TDOs are homo-tetrameric enzymes with 35–45 kD monomers and are well conserved from bacteria to humans (Supplementary Fig. 1). In comparison, IDOs are monomeric enzymes and the sequence conservation between TDO and IDO is much weaker, with 16% sequence identity between hTDO and human IDO1 (hIDO1) (Supplementary Fig. 1). While the properties of TDO have been studied extensively^1^,^2^, the detailed dioxygenase mechanism remains poorly understood.

Crystal structures of bacterial TDOs^25^,^26^, *Drosophila* TDO (DmTDO)^27^, apo hTDO^28^, and hIDO1^29^ show that the overall folds and the active sites of TDO and IDO are highly similar, despite their weak sequence conservation. The binding mode of *L*-Trp to *Xanthomonas campestris* TDO (XcTDO, with 34% sequence identity to hTDO, Supplementary Fig. 1) is known^25^. However, it is unclear how O_2_ is positioned with respect to *L*-Trp in the active site, which is essential for comprehending the dioxygenase mechanism. In addition, the binding mode of the reaction product NFK is not known either.

## Results and Discussion

### Structure of hTDO in complex with *L*-Trp and O_2_

**Figure 1:**
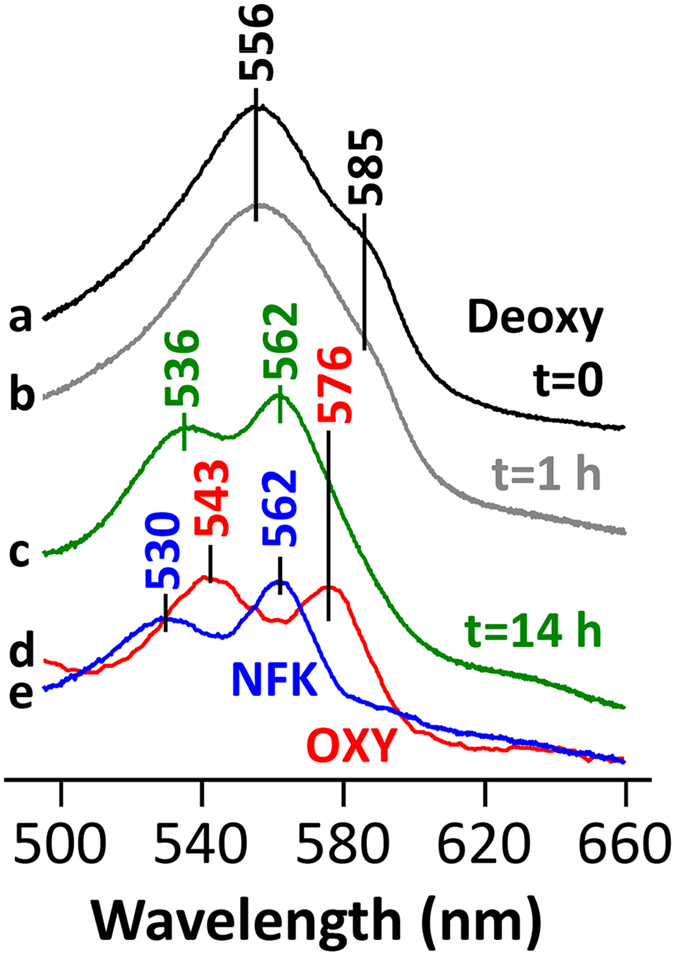
Absorption spectra of hTDO oxygen intermediates. (**a**) Spectrum of a ferrous hTDO crystal in complex with *L*-Trp, before exposure to O_2_-saturated solution (black). The spectrum has a band at 556 nm and a shoulder at ~585 nm, which is characteristic for a five-coordinate high spin heme. (**b**) Spectrum of the same ferrous hTDO crystal in complex with *L*-Trp, after 1h exposure to O_2_ (gray). (**c**) Spectrum of the same ferrous hTDO crystal in complex with *L*-Trp, after overnight exposure to O_2_ (~14 h) (green), with two bands at 536 and 562 nm, indicating the formation of an oxygen intermediate. (**d**) Typical spectrum of the hTDO-Trp-O_2_ ternary complex obtained by mixing of ferrous hTDO-Trp with air-saturated solution in a stopped-flow system (red). (**e**) Difference spectrum, (**c**,**d**) which likely represents the spectrum of NFK-bound ferrous hTDO (blue). The double peak feature of this spectrum indicates a six-coordinate low spin heme, consistent with the crystallographic observation that NFK is coordinated to the heme iron via its formyl group as the 6^th^ ligand. This spectrum cannot be obtained by mixing hTDO and NFK due to the transient nature of this complex.

To define the binding mode of O_2_ with respect to *L*-Trp and to obtain direct molecular insight into the catalytic mechanism, we prepared crystals of ferrous hTDO in complex with *L*-Trp under anaerobic conditions and then exposed them to an O_2_-saturated solution at room temperature. The reaction between O_2_ and Trp in the active site was monitored by microscopic spectroscopy. We observed a large change in the absorption spectrum of the crystal after overnight exposure to O_2_ (Fig. 1), indicating O_2_ binding and dioxygenation reaction. The crystal was then flash-frozen for X-ray diffraction data collection. The final atomic model, at 2.5 Å resolution, has excellent agreement with the crystallographic data and the expected bond lengths, bond angles, and other geometric parameters (Table 1). The majority of the residues (92%) are in the favored region of the Ramachandran plot. Several other data sets were collected on crystals exposed to O_2_ using similar protocols, and comparable electron density was observed in the active sites of these crystals as well. In contrast, no electron density for O_2_ was observed in crystals mounted directly in the glove box, and the structure of hTDO in this binary complex with *L*-Trp is mostly the same as that obtained following O_2_ exposure (Table 1).

**Figure 2:**
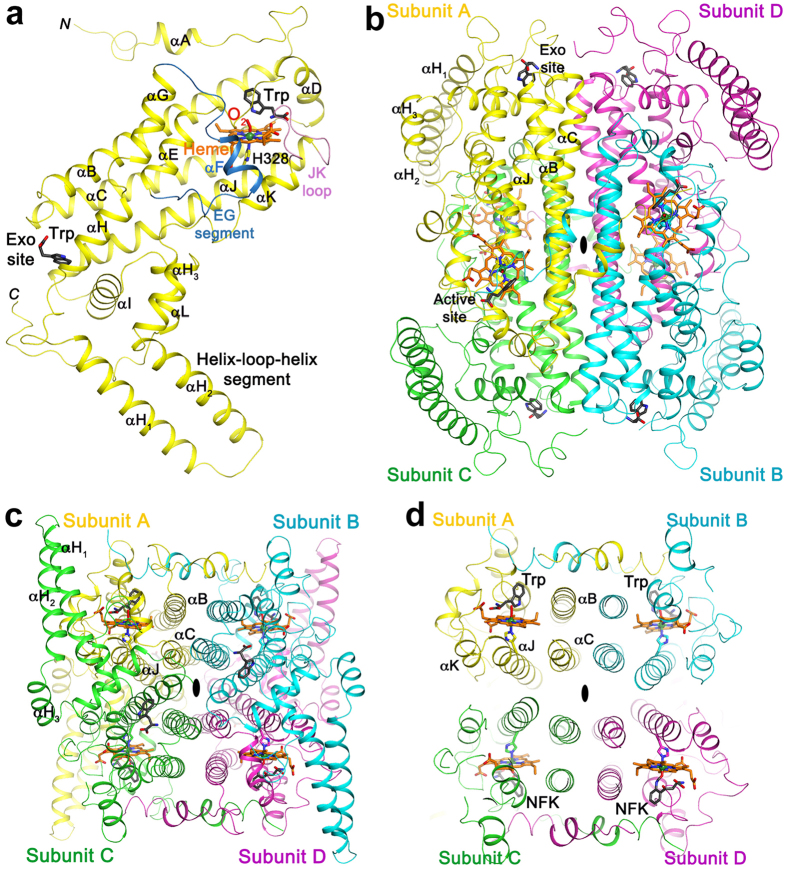
The structure of human TDO (hTDO). (**a**) Schematic representation of the structure of the hTDO monomer in a ternary complex with *L*-Trp and O_2_. Heme (in orange), *L*-Trp in the active site and the exo site (black), and oxygen (red) are shown as stick models. (**b**) Schematic representation of the hTDO tetramer, viewed down the two-fold axis (black oval) relating subunits A and B. The four subunits (A, B, C, and D) are colored in yellow, cyan, green and magenta, respectively. (**c**) Schematic representation of the hTDO tetramer viewed down the two-fold axis (black oval) relating subunits A and D. (**d**) A thin section of the structure of the hTDO tetramer, centered around the four hemes in the active sites, showing the three helices in each subunit that are in the tetramer interface. All structure figures were produced with the program PyMOL (www.pymol.org).

The hTDO monomer has an all α-helical structure, and the helices are named αA through αL, as in XcTDO (Fig. 2a, Supplementary Fig. 1). Three long helices (αB, αC, and αJ), with 6–10 turns each, are at the center of the hTDO tetramer interface (Fig. 2b–d). Two additional helices, αE and αH, combine to form another long helix, producing a four-helical bundle in each subunit (Fig. 2a, Supplementary Fig. 2). The heme is located at one end of this four-helical bundle, with the proximal His328 ligand coming from the C-terminal region of helix αJ (Fig. 2a). A helix-loop-helix segment (αH_1_–αH_2_) is located near the other end of this bundle (Fig. 2a), which is also present in DmTDO but not XcTDO (Supplementary Fig. 3).

### Binding modes of the substrates *L*-Trp and O_2_

**Figure 3:**
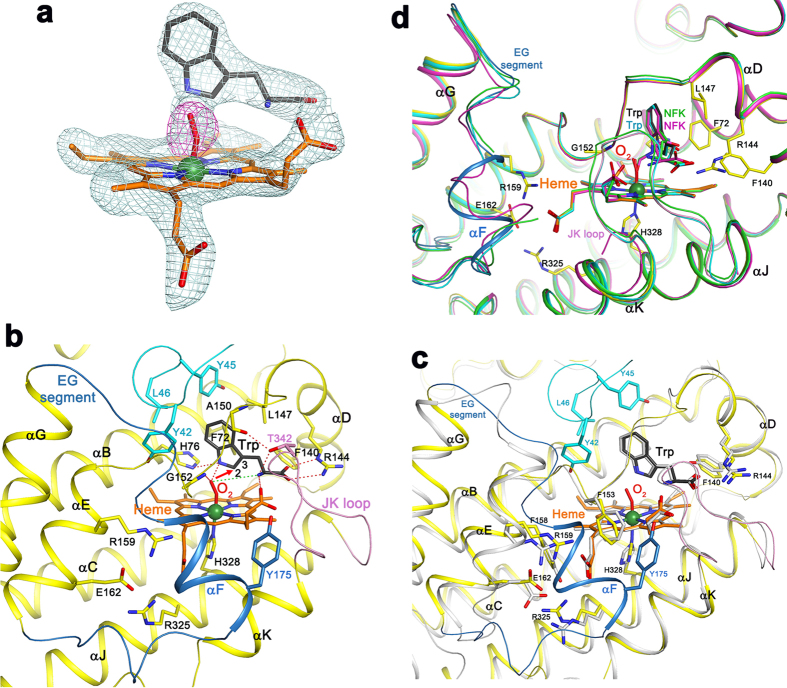
Molecular basis for substrate recognition by hTDO. (**a**) Omit F_o_–F_c_ map (light blue) at 2.5 Å resolution for heme, *L*-Trp and O_2_ in the active site of subunit A, contoured at 3σ. Omit F_o_–F_c_ map for O_2_ (magenta), contoured at 5σ. (**b**) Schematic drawing showing the active site of hTDO in the ternary complex with *L*-Trp and O_2_. The segment in cyan is from subunit B of the tetramer, which forms a part of the binding pocket for the Trp side chain. The ionic interaction between the terminal oxygen atom and the ammonium ion of *L*-Trp is indicated by the dashed line in green. The attack of O_2_ on the C_2_ atom of *L*-Trp is indicated with the red arrow. (**c**) Overlay of the active site structures of hTDO (in color) and XcTDO (in gray). Residues Tyr42, Tyr45 and Leu46 from the N-terminal segment of the neighboring subunit are shown in cyan. (**d**) Overlay of the active site structures of the four subunits of hTDO. Conformational differences for the EG segment and the disordering the JK loop in two subunits are visible.

The crystallographic analysis revealed that the active sites of the four subunits of the hTDO tetramer are in different states in terms of the catalytic conversion of *L*-Trp, giving us several snapshots along the reaction coordinate. Clear electron density was observed for *L*-Trp and O_2_ in subunits A (Fig. 3a) and B, allowing the definition of the relative regioorientation of the two substrates prior to the reaction for the first time. The O_2_ substrate is coordinated to the heme iron as the sixth ligand. The Fe-O-O moiety is slightly bent (Fig. 3b), with a Fe-O-O angle of ~150°. The angle is significantly larger than that expected for a typical ferrous iron bound neutral dioxygen (∠Fe-O-O = 120°)^30^, consistent with the [Fe^3+^-O_2_^−^] electronic configuration revealed by earlier resonance Raman studies^31^. The terminal oxygen atom is situated next to the plane of the *L*-Trp indole ring, close to its C_2_ (2.9 Å distance) and N_1_ (2.5 Å) atoms. Intriguingly, the electron density for the terminal oxygen atom is connected to these two atoms of the indole ring in subunit A, although the origin of this connection is not clear and this connection is much weaker in subunit B. We tried different conformational states for *L*-Trp but none of them could satisfactorily explain the density connecting O_2_ to the indole. The terminal oxygen also has strong hydrogen-bonding interactions with the main-chain amide of Gly152 (2.5 Å) in the DE loop (connecting helices αD and αE). This oxygen is 4.4 Å from the main-chain ammonium ion of the *L*-Trp substrate, although earlier experimental and QM/MM studies suggested that they are within hydrogen-bonding distance^32^,^33^,^34^. These interactions strategically position O_2_ next to *L*-Trp for the catalysis.

The recognition of the *L*-Trp substrate by hTDO (Fig. 3b) is similar to that by XcTDO^25^ (Fig. 3c), as most of the residues in the active site region of the two enzymes are conserved (Supplementary Fig. 1). His76 is hydrogen-bonded to the N_1_ atom of *L*-Trp, although this interaction is not essential for TDO catalysis^35^,^36^ and this residue is replaced by Ser in IDO (Supplementary Fig. 1). The JK loop, with a β-reverse turn structure at its tip formed by a highly conserved GTGG motif (Supplementary Fig. 1), covers the active site (Fig. 3b). The hydroxyl group of the Thr side chain in this motif is hydrogen-bonded to the ammonium ion of *L*-Trp and the main-chain carbonyl oxygen of Ala150, which may also help organize the active site for catalysis.

### Binding mode of the product NFK

**Figure 4:**
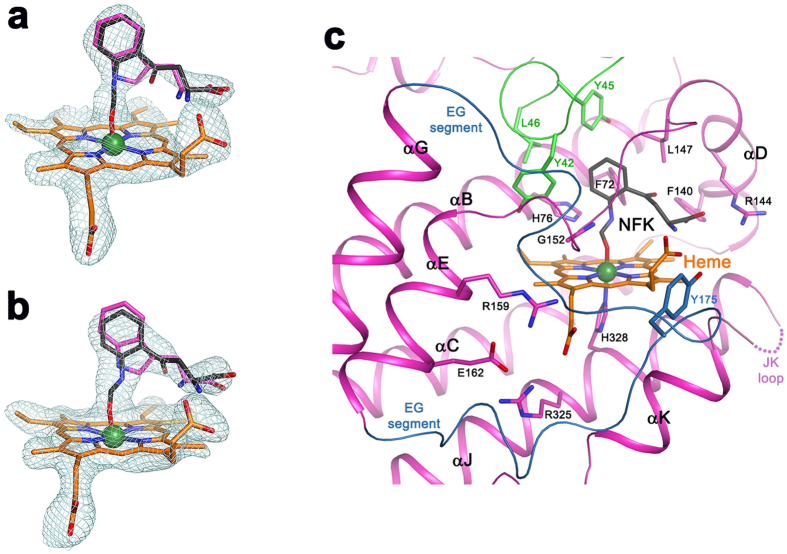
Molecular basis for NFK recognition by hTDO. (**a**) Omit F_o_–F_c_ map at 2.5 Å resolution for heme and NFK in the active site of subunit C, contoured at 2.5σ. The binding mode of Trp in subunit A is shown as a reference. (**b**) Omit F_o_–F_c_ map at 2.5 Å resolution for heme and NFK in the active site of subunit D, contoured at 2.5σ. (**c**) Schematic drawing showing the active site of hTDO in the binary complex with NFK in subunit D, where the JK loop is disordered (dotted curve).

The electron density in the active site of subunit C (Fig. 4a) and subunit D (Fig. 4b) is consistent with the product NFK, allowing us to define for the first time the binding mode of this product to TDO. The pyrrole ring of the Trp side chain has opened, but good electron density was observed only for the remaining six-membered ring and its *N*-formyl group, which is coordinated to the heme iron. In contrast, the indole ring of *L*-Trp does not fit well into the density, and the ring cannot explain the strong density connection to the iron (Fig. 4a,b). The JK loop is ordered in subunit C but disordered in subunit D (Fig. 4c). The rest of the structures of the two subunits are similar to each other (Supplementary Fig. 2). The observation that two subunits of the tetramer are trapped in the O_2_ and Trp bound state while the other two are in the product-bound state seems to indicate that the dioxygenase reaction is cooperative, but so far no cooperativity is observed in free solution reactions of hTDO. Nonetheless, EPR and Mössbauer studies of a bacterial TDO do support that the four heme sites in TDO are not equivalent^37^.

### Implications for hTDO catalytic mechanism

**Figure 5:**
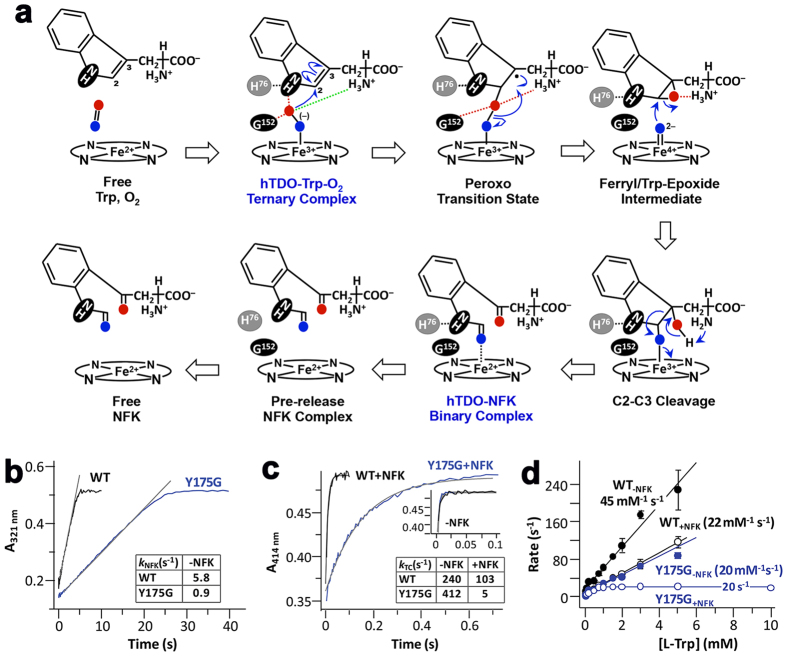
Catalytic mechanism of hTDO. **(a**) A two-step, ferryl-based mechanism for the dioxygenation of *L*-Trp. The two complexes observed in the current crystal are labeled in blue. The electrostatic interaction between the terminal oxygen of O_2_ and the main-chain ammonium ion of *L*-Trp is indicated with the dashed lines in green. Modified from earlier publications^31^,^32^,^33^. **(b**) Stopped-flow kinetics showing that the multiple turnover activity of the enzyme is reduced by the Y175G mutation. The production of NFK was monitored by absorbance at 321 nm. **(c**) Stopped-flow kinetics showing that the presence of NFK (8 mM) retarded Trp-O_2_ binding to the deoxy enzymes to form the ternary complexes, especially for the Y175G mutant. TC: ternary complex, monitored by absorbance at 414 nm. The inset shows the kinetic traces obtained in the absence of NFK. **(d**) Trp binding kinetics and its retardation by NFK. The Trp binding rate of the Y175G mutant is linear with [Trp], leading to a *k*_on_ of 20 mM^−1^s^−1^. In the presence of 8 mM NFK, the same *k*_on_ was observed at low [Trp]; as the [Trp] was raised above 1 mM, the rate leveled off at 20 s^−1^, as the reaction was rate-limited by NFK release. The data in (**b**) and (**c**) were taken from Supplementary Figs 4 and 5.

The crystallographic analyses have allowed us to directly visualize both the substrate and product complexes of hTDO. The structural data, together with earlier spectroscopic and computational studies^31^,^32^,^33^, support a two-step ferryl-based dioxygenation mechanism (Fig. 5a). The first step of the reaction is initiated by radical addition of the heme-iron-bound dioxygen to C_2_ of *L*-Trp to generate a ferryl and Trp-epoxide intermediate, via a 2-indolenylperoxo transition state. An alternative, electrophilic addition of O_2_ to *L*-Trp in the first step of the reaction has also been proposed^38^,^39^,^40^, but QM/MM calculations favor radical addition mechanism^31^,^32^,^33^. In the second step, protonation of the epoxide by the ammonium ion of *L*-Trp opens the epoxide ring and triggers the addition of the ferryl-oxygen to C_2_, ultimately leading to the breakage of the C_2_–C_3_ bond and the formation of the NFK product. The formyl group of NFK is coordinated to the iron atom after the reaction. The disordering of the JK loop allows NFK to diffuse out of the active site, thereby enabling the binding of a new *L*-Trp for the next round of the reaction.

Besides the disordering of the JK loop in subunit D, the structures of the four subunits in the immediate active site region are highly similar to each other (Fig. 3d). On the other hand, large conformational differences in the segment connecting αE and αG (EG segment) are observed in the four subunits (Fig. 3d). This segment contains a short helix (αF) in subunit A (Fig. 3b), but is partly disordered in subunits B and C (Fig. 3d), and is a loop in subunit D (Fig. 4c), where its conformation is partly stabilized by crystal packing. The Tyr175 side chain in this segment is positioned near the JK loop in subunit A (Fig. 3b), but in subunit D it would clash with the JK loop if the loop were ordered (Fig. 4c). In comparison, the EG segment in XcTDO is positioned further away from the active site due to a deletion in this region (Fig. 3c and Supplementary Fig. 1). On the other hand, the EG segment, as well as the JK loop and helix αK are disordered in the apo hTDO structure^28^ (Supplementary Fig. 3), due to the absence of the heme and substrates.

To assess the functional importance of the EG segment, we created the Y175G mutant and compared its activity to that of the wild-type (WT) enzyme with stopped-flow measurements (Supplementary Figs 4 and 5). As summarized in Fig. 5, the mutation led to a 6-fold slower multiple turnover velocity (Fig. 5b). In addition, pre-incubation of the Y175G mutant with 8 mM NFK retards the formation of the ternary complex by ~100-fold (Fig. 5c) and impedes Trp binding (Fig. 5d). Overall, the data suggest that the EG segment in hTDO plays a critical role in promoting NFK release, thereby allowing Trp binding during multiple turnover.

### An exo site for *L*-Trp binding in hTDO

**Figure 6:**
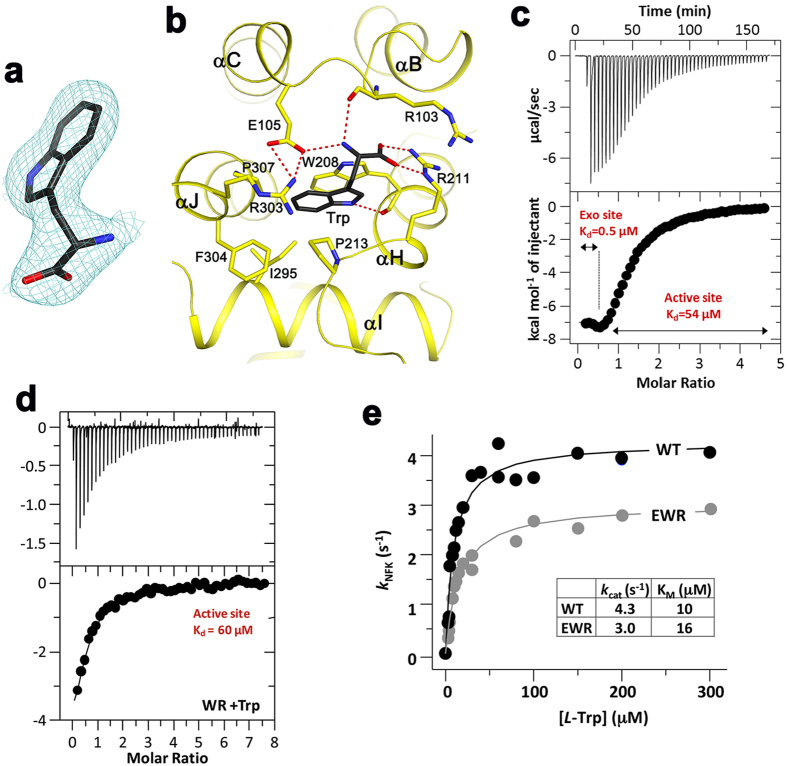
A novel exo site for *L*-Trp in hTDO. **(a**) Omit F_o_–F_c_ map at 2.5 Å resolution for *L*-Trp in the exo site of subunit A, contoured at 4.5σ. **(b**) Schematic drawing showing the interactions between *L*-Trp and the exo site. Hydrogen-bonding and ionic interactions are indicated with the dashed lines in red. **(c**) ITC data for wild-type hTDO demonstrating the presence of two *L*-Trp binding sites, with association constants of 2.06 ± 1.34 × 10^6 ^M^−1^ (*K*_d_~0.5 μM) and 1.84 ± 0.04 × 10^4 ^M^−1^ (*K*_d_~54 μM). (**d**) Representative ITC titration of the WR exo site mutant with Trp in the ferric state, showing binding only to the active site. (**e**) Michaelis-Mikenten plots of the wild type (WT) and EWR mutant reactions showing that the exo site does not significantly perturb the *k*_cat_ and *K*_m_ of the enzyme.

The structure revealed that a second *L*-Trp molecule is bound to each hTDO subunit, with well-defined electron density (Fig. 6a). This exo site is located at the other end of the four-helical bundle and is ~42 Å from the active site (Fig. 2a). The side chain of this *L*-Trp is sandwiched between Trp208 (helix αH) and Pro213, and is in direct contact with several other hydrophobic residues (Fig. 6b). The main-chain carboxylate group has bidentate ion-pair interactions with the side chain of Arg211 (αH). The ammonium ion of *L*-Trp is positioned near the main-chain carbonyl of Arg103 and the side chain of Glu105, which also has ion-pair interactions with Arg303.

Residues forming this novel exo site are well conserved among tryptophan-auxotrophic TDOs, but they are generally poorly conserved in other orthologs (Supplementary Fig. 1). This binding site does not exist in XcTDO, due to side-chain substitutions as well as main-chain conformational differences. Nonetheless, a second binding site for *L*-Trp was observed in XcTDO at the tetramer interface^25^ (Supplementary Fig. 3), while the exo site in hTDO is located within each subunit.

We next carried out isothermal titration calorimetry (ITC) experiments to verify the presence of two *L*-Trp binding sites in hTDO. Two transitions were observed, corresponding to association constants of 2.06 ± 1.34 × 10^6 ^M^–1^ (*K*_d_~0.5 μM) and 1.84 ± 0.04 × 10^4 ^M^–1^ (*K*_d_~54 μM) (Fig. 6c), which we assigned to the exo site and the active site, respectively. To confirm this assignment, we generated a double mutant W208V/R211L (referred to as the WR mutant hereafter) and showed that the mutation abolished the high affinity site without significantly perturbing the low affinity site (Fig. 6d). Overall, the ITC data revealed that the exo site exhibits 100-fold higher affinity for *L*-Trp than the active site, and that the WR double mutation is sufficient to disrupt *L*-Trp binding to the exo site.

We also examined whether the exo site has any effect on the catalytic activity of hTDO. While it might be expected that the exo site can regulate hTDO catalysis indirectly by modulating the organization of the four helical bundle (Fig. 2a), our kinetic data indicate that a triple mutant, E105L/W208V/R211L (EWR mutant), had only a minor defect in catalysis as compared to the wild-type enzyme (Fig. 6e).

### The exo site regulates hTDO cellular stability

Mouse TDO (mTDO) is one of the most rapidly degraded hepatic proteins, with a half-life (t_1/2_) of ~2.5 h, versus a mean t_1/2_ of ~2–3 days for total liver protein^41^. This unusually short lifespan is consistent with the large number (15) of mTDO ubiquitination sites^42^, which probably enables its rapid removal via ubiquitin (Ub)-dependent proteasomal degradation (UPD). A second Trp-binding site in mTDO was previously proposed based on *in vivo* and *in vitro* studies^41^. In addition, it was shown that binding of *L*-Trp, or its α-methyl analog (αMTrp), to this site not only stabilizes the enzyme against heat, urea or proteases, but also reduces its hepatic degradation.

**Figure 7:**
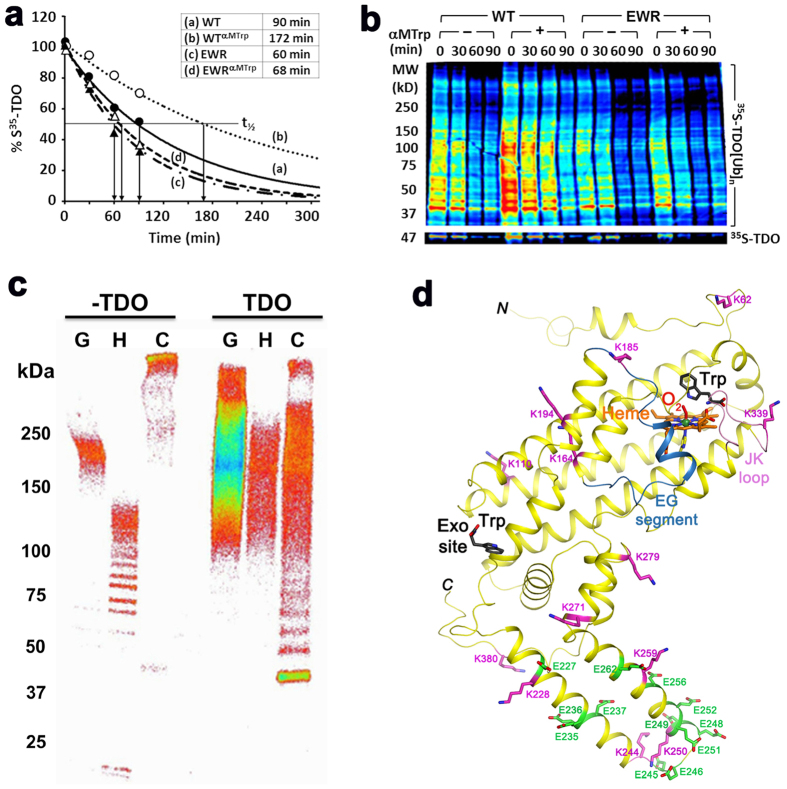
The exo site is required for *L*-Trp-elicited hTDO protein stabilization against UPD. **(a**) ^35^S-pulse-chase analyses of hTDO protein degradation in HepG2 cells, showing αMTrp-stabilization of the WT hTDO protein but not its EWR mutant with the disrupted exo site. **(b**) SDS-PAGE/fluorographic analyses of the ^35^S-hTDO species pulled-down from the pulse-chase experiments. A shorter time-exposure of the parent ^35^S-TDO species (47 kD) in the same gel is shown in the bottom panel. Color code wheel: Red > orange > yellow > green > blue > indigo > violet. αMTrp greatly stabilizes hTDO by reducing the degradation of its parent and ubiquitinated species in cells expressing the WT protein, but not the EWR mutant. (**c**) *In vitro* ubiquitination of hTDO by three E2/E3 systems – G: Ubc7/gp78; H: Ubc7/Hrd1; C: UbcH5a/CHIP/Hsc70/Hsp40. Experimental details of the ubiquitination reactions are described in ref. 43. Color code wheel: Violet > indigo > blue > green > yellow > orange > red > . The comparative studies demonstrate that among the three complexes tested, the Ubc7/gp78 complex is the most efficient ubiquitination system for hTDO. (**d**) Structure of hTDO monomer showing the 13 ubiquitination K-sites (magenta) and the negatively charged glutamate residues (green) in the helix-loop-helix segment. Two additional K-sites identified in this study, K17 and K37, are not shown. K17 is not included in the expression construct, and K37 is located in a disordered region.

We hypothesized that the exo site observed in the current hTDO structure is equivalent to the second site functionally identified in mTDO and that binding of *L*-Trp to the exo site stabilizes hTDO against UPD, thereby regulating its biological lifespan. To test this hypothesis, we first carried out ^35^S-pulse-chase analyses of a full-length His_**6**_-tagged hTDO protein expressed in a human liver HepG2 cell culture. We found that the inclusion of αMTrp, which primarily binds to the exo site (as its α-methyl group has steric clashes with the heme in the active site), in the culture media indeed increased the *in vivo* t_1/2_ of hTDO protein by ~2-fold (from 90 to 172 min), based on total radioactivity (Fig. 7a) as well as SDS-PAGE/fluorographic analyses of the pulled-down parent (47 kD) and ubiquitinated (>56 kD) hTDO species (Fig. 7b). In contrast, the EWR mutant was not stabilized by αMTrp, confirming that the stabilization is due to αMTrp binding to the exo site. We used αMTrp rather than *L*-Trp in these experiments as HepG2 cells require aerobic culture for viability, and exogenous *L*-Trp is readily consumed by the over-expressed hTDO under these conditions. Marked hTDO stabilization was also observed in HepG2 cells upon inclusion of the proteasomal inhibitor MG132 (10 μM), thereby attesting to its cellular UPD (data not shown). Together, these data verify that UPD is a major pathway for cellular hTDO disposal and that αMTrp/*L*-Trp binding to the exo site can enhance its intracellular stability by reducing its UPD.

To identify the hTDO ubiquitination sites, we incubated the enzyme with two E2/E3 Ub-ligase complexes that are major participants in the UPD of some hepatic proteins^43^,^44^ and identified 15 ubiquitinated Lys residues by Ub-remnant profiling and LC-MS/MS analyses (Table 2). While similar proteomic analyses of mouse liver extracts identified ten of these sites in mTDO previously^42^, 5 of the sites identified here (K17, K110, K185, K194 and K259) are entirely new (Table 2). In addition, we found that the three E2/E3 complexes have different hTDO-ubiquitination efficiencies: Ubc7/gp78 > UbcH5a/CHIP > Ubc7/Hrd1 (Fig. 7c).

Molecular recognition of hepatic cytochromes P450 by these E2/E3 Ub-ligases involves electrostatic interactions between positively charged E2/E3-domains and negatively charged surface clusters of phosphorylated Ser/Thr and acidic Asp/Glu-residues. Lys residues within such clusters in P450 were shown to be predominantly targeted for ubiquitination^44^. The 15 hTDO ubiquitination sites are distributed throughout the surface of the monomer (Fig. 7d) and tetramer. Intriguingly, the helix-loop-helix segment of hTDO is rich in negatively-charged Glu residues and 5 of its 15 identified ubiquitination sites are located within this region (Fig. 7d). Conceivably, this segment is important for the molecular recognition of hTDO by these E2/E3 complexes, and the subsequent ubiquitination of Lys residues in this segment is required for UPD. As the exo site is located close to this helix-loop-helix segment (Fig. 2a), this offers a plausible molecular mechanism for this site to regulate hTDO ubiquitination and UPD. Our studies have thus provided novel mechanistic insights into the cellular regulation of hTDO degradation by its *L*-Trp substrate, which may have important implications for the role of this enzyme in human diseases. They also offer invaluable structural basis for future QM/MM studies to further refine the proposed ferryl-based dioxygenation mechanism.

## Methods

### Protein expression and purification

Expression of hTDO was performed as described elsewhere^4^,^36^. To ensure homogeneity of the sample, the protein was oxidized with potassium ferricyanide and immediately loaded on a G25 column pre-equilibrated with 50 mM Tris (pH 7.4) and 150 mM NaCl. The protein collected was flash frozen and stored in − 80 °C until use.

### Protein crystallization

The binary complex of hTDO was crystallized using the under-oil microbatch method under anaerobic conditions inside a glove box at 18 °C. 3 μl of hTDO (45 mg/ml) in 50 mM Tris (pH 8.0), containing 150 mM NaCl and 10 mM *L*-Trp, was reduced with 2-fold molar excess of sodium dithionite. It was then mixed with 3–6 μL of a precipitant solution, containing 50 mM sodium citrate (pH 5.6), 5% (w/v) PEG 3350, and 2% (w/v) Tacsimate (pH 5). Crystals were harvested after 3–5 days. The optical absorption spectra were taken to ensure the crystals were in a fully reduced deoxy state.

### Microscopic spectroscopy

Crystals of the Trp-bound binary complex were soaked in an O_2_-saturated precipitant solution supplemented with 20% (v/v) ethylene glycol at room temperature. The in-crystal reactions were monitored with a customized Raman microscope system (Labram HR from Horiba Jobin Yvon). Very little change in the spectrum was observed within an hour. Overnight (~14 h) incubation led to a new spectrum indicating the formation of an oxygen intermediate. The intermediate crystals were flash-frozen in liquid nitrogen for structure determination. The crystal was annealed at the beamline before data collection due to ice accumulation.

### Data collection and structure determination

X-ray diffraction data were collected at the X4A and X4C beamlines of National Synchrotron Light Source (NSLS). The diffraction images were processed with the program HKL^45^, and the data processing statistics are summarized in Table 1.

The structure of hTDO was determined by the molecular replacement method, as implemented in the program MOLREP^46^, using the structure of XcTDO tetramer (PDB entry 2NW8) as the search model^25^. The atomic models were built with the program XtalView^47^, and the structure refinement was carried out with CNS^48^ and PHENIX^49^. The refinement statistics are summarized in Table 1. Coordinates and structure factors have been deposited in the Protein Data Bank with accession codes 5TI9 and 5TIA.

### Mutagenesis

The mutants were made with the QuikChange kit (Stratagene) and verified by sequencing. The mutant proteins were expressed and purified following the same protocol as that for the wild-type protein.

### Stopped-flow measurements

The experiments were performed by either mixing deoxy ferrous enzymes (final concentration ~0.5 or 3 μM) with air-saturated buffer or mixing ferric enzymes with buffer containing different concentrations of L-Trp in a π* 180 system from applied Photophysics Ltd (Leatherhead, Surrey, UK).

The deoxy ferrous enzyme was prepared by stoichiometrically titrating N_2_-purged ferric enzyme with dithionite. All the solutions were prepared in pH 8 Tris buffer (50 mM), containing 150 mM NaCl and a desired amount of Trp. The temperature was controlled at 25 °C by a circulating water bath (Neslab RTE-9DD). For steady-state activity measurements, the formation of the product, NFK, was monitored at 321 nm (ε = 3750 M^−1^cm^−1^) as a function of time. The initial linear velocity of the reaction was plotted as a function of substrate concentration and fitted with the Michaelis-Menten equation using Origin 6.1 software (Microcal Software, Inc., MA, USA).

### Isothermal titration calorimetry (ITC) measurements

The Trp affinities were measured in a Microcal VP-ITC (Northhampton, MA) in an anaerobic glove box. The ITC was calibrated using the built-in electrical calibration check. The ferric WT hTDO solution (0.2 mM) in the reaction cell and the Trp solution (3.5 mM) in the syringe were prepared in the same buffer in ddH2O (pH 8 100 mM Tris containing 150 mM NaCl). All solutions were pre-purged with nitrogen gas, to avoid turnover. Following thermal equilibrium at 20 °C, an initial 600 s delay and a single 2.0 μl titrant injection, 10 serial injections of 4 μl, followed by 40 serial injection of 6 μl Trp solution, were done at an interval of 300 s into the stirred sample cell (1.4 mL) containing the ferric hTDO complex at a stirring rate of 155 rpm. A reference power of 15 μcal/s was used. The heat associated with each titration peak was integrated and plotted against the respective molar ratio of Trp/hTDO. Similar procedure was used for the measurement of the W208V/R211L mutant (0.1 mM), except that following thermal equilibrium at 20 °C, an initial 600 s delay and a single 2.0 μl titrant injection, 47 serial injections of 6 μl Trp solution were done at an interval of 450 s into the stirred sample cell. Data were analyzed using nonlinear least-squares curve fitting in Origin7.0 (OriginLab Corp., Northampton, MA) using the standard one-binding site model.

### ^35^S-*L*-Met/Cys-pulse-chase analyses

HepG2 cells were transfected with the pcDNA6-hTDO-(His)_6_ or pcDNA6-EWR-(His)_6_ vector and grown to confluency in DMEM in 6-well-plates for 48 h. The medium was then removed and replaced with methionine/cysteine-free DMEM containing 2.5 mM α-methyltryptophan (αMTrp) for 1 h. Subsequently, each well was pulsed with 75 μCi of ^35^S-L-Met/Cys for 1 h. Each ^35^S-labeled culture was then washed twice with ice-cold PBS containing 0.2 mM methionine and 1.4 mM cysteine, followed by DMEM containing 2.5 mM αMTrp, 5 mM cold methionine and cysteine, and further incubated for 0, 30, 60 and 90 min at 37 °C. The cells harvested at each time point were lysed in Cell-Signaling Lysis buffer, containing general protease and phosphatase inhibitors, and N-ethylmaleimide (10 mM) to inhibit deubiquitinases. The lysates were centrifuged at 10,000 g at 4 °C for 10 min to remove insoluble cell debris. The protein concentration was determined by the bicinchoninic assay (BCA). Lysate protein (200 μg) was then diluted (1:4, v:v) in Dynabead pull-down buffer and mixed with Dynabeads (50 μl). The mixture was incubated at 4 °C with rotation overnight. The Dynabeads-His_6_-tagged hTDO protein complexes were then collected using a magnetic stand and washed five times with Dynabead-washing buffer. The His_6_-tagged hTDO proteins were eluted by heating the complexes for 5 min in an SDS-PAGE sample-loading buffer (40 μl, 62.5 mM pH 6.8 Tris buffer containing 25% (v/v) glycerol, 10% (w/v) SDS, 5% (v/v) β-mercaptoethanol and 0.01% (w/v) bromophenol blue). The radioactivity of a 10 μl aliquot was monitored in 4 ml of Ecolume using a Beckman LS3801 liquid scintillation counter. The radioactivity of the eluates was used for the pulse-chase ^35^S-TDO-degradation analysis. Another 30 μl-aliquot of the eluate (containing parent TDO protein and its ubiquitinated species) was subjected to SDS-PAGE (4–15%). The gels were dried and subjected to fluorography with Typhoon scanning.

### *In vitro* hTDO ubiquitination by 3 different E2/E3 Ub-ligase systems

Purified recombinant hTDO (400 pmol) was incubated in each of the three functionally reconstituted E2/E3 systems in a final volume of 50 μl, and the reactions initiated with an ATP-regenerating system as described previously^43^,^44^,^50^. The reaction mixtures were incubated at 30 °C for 60 min. Aliquots (30 μl) of each reaction mixture were then subjected to SDS-PAGE and Western immunoblotting analyses with subsequent 5-min exposure to electrochemiluminescent (ECL) substrate for pico-detection, as described^44^,^50^. The film was developed and visualized using a Typhoon scanner in the chemiluminescence mode.

### Identification of hTDO K-ubiquitination sites

Ubiquitinated hTDO proteins generated as described above were combined and precipitated with 2-volumes of ice-cold acetone at −20 °C, overnight. The pellets were redissolved in 50 mM ammonium bicarbonate (ABC) solution containing 8 M urea and reduced with tris (2-carboxyethyl) phosphine (TCEP, 10 mM) and then alkylated with chloroacetamide (20 mM) at room temperature in the dark and diluted with the same ABC solution to a final concentration of <2 M urea followed by a combined lysyl endoprotease C (Lys-C)/trypsin digestion with a 1:25 enzyme:protein mass ratio, at 37 °C for 16–18 h. The digested peptides were desalted and extracted with a Sep-Pak C18 classic cartridge (Waters Inc.) and subjected to anti-KGG antibody (PTMScan Ubiquitin Remnant Motif kit, Cell Signaling) pull-down to enrich the ubiquitinated peptides before LC-MS/MS analyses. Ubiquitinated samples were analyzed on an LTQ-Orbitrap Velos mass spectrometer (Thermo Scientific) equipped with a nanoAcquity UPLC system (Waters). Peptides were resuspended in 0.1% formic acid and separated in an Easy-Spray column (Thermo, PepMap, C18, 3 μm, 100 Å, 75 μm × 15 cm) using a chromatographic system with a linear gradient from 2% solvent A (0.1% formic acid in water) to 35% solvent B (0.1% formic acid in acetonitrile) at 300 nL/min over 35 min. MS precursor spectra were measured in the Orbitrap from 300–2000 *m/z* at 30,000 resolving power, selected and dissociated by higher energy collisional dissociation (HCD) for MS/MS analyses as described^44^,^50^. The MS/MS data were searched against the SwissProt database using the in-house Protein Prospector search engine (UCSF), with a concatenated database consisting of normal and randomized decoy databases. In addition to common modifications such as methionine oxidation and protein N-terminal acetylation, GlyGly (Uncleaved K), LeuArgGlyGly (Uncleaved K) modifications were considered for ubiquitination in the database search. False discovery rate (FDR) for ubiquitination was estimated to be 1% corresponding to the maximum expectation values of 0.01. Peptides with expectation values of 0.01 or less were accepted.

## Additional Information

**How to cite this article**: Lewis-Ballester, A. *et al.* Molecular basis for catalysis and substrate-mediated cellular stabilization of human tryptophan 2,3-dioxygenase. *Sci. Rep.* **6**, 35169; doi: 10.1038/srep35169 (2016).

## Supplementary Material

Supplementary Information

## Figures and Tables

**Table 1:** Data collection and refinement statistics.

	Crystal exposed to O_2_ (Ternary complex with *L*-Trp and O_2_ and binary complex with NFK)	*L*-Trp ferrous binary complex (anaerobic)
**Data collection**		
Space group	*P*2_1_2_1_2	*P*2_1_2_1_2
Cell dimensions		
*a*, *b*, *c* (Å)	143.6, 154.0, 87.8	144.4, 153.6, 88.2
*α*, *β*, *γ* (°)	90, 90, 90	90, 90, 90
Resolution (Å)	48.3–2.5 (2.54–2.5)*	35.1–2.44 (2.54–2.44)*
*R*_merge_	0.062 (0.552)	0.107 (0.792)
*I*/σ*I*	37.3 (4.3)	32.0 (3.3)
Completeness (%)	99.9 (98.5)	100 (100)
Redundancy	6.8 (5.8)	8.5 (7.9)
**Refinement**		
Resolution (Å)	34–2.5 (2.53–2.5)	35.1–2.44 (2.47–2.44)
No. reflections	68,151	72,907
*R*_work/_*R*_free_	15.9/23.0	16.8/23.0
No. atoms	12,070	12,061
Protein	11,342	11,319
Ligand/ion	288	268
Water	436	474
B-factors		
Protein	45.4	44.8
Ligand/ion	48.6	43.6
Water	45.3	48.3
R.m.s deviations		
Bond lengths (Å)	0.011	0.008
Bond angles (°)	1.3	1.1

One crystal was used for each data collection.

*Highest resolution shell is shown in parenthesis.

**Table 2:** List of hTDO sites ubiquitinated by two E2/E3 systems, Ubc7p/gp78 and UbcH5a/CHIP^#^.

No.	Site	Peptide sequence	m/z	z	ppm	Score	Expect val.
1a*	17	K(GlyGly)LPVEGSEEDK	448.8929	3	−0.003	43.0	8.9e-9
1b*	17	K(LeuArgGlyGly)LPVEGSEEDK	538.6210	3	−0.54	29.4	3.3e-6
2	37	ASK(GlyGly)GGLIYGNYLHLEK	626.3360	3	1.00	46.0	1.4e-5
3	62	VLNAQELQSETK(GlyGly)GNK	886.9619	2	1.60	52.5	2.2e-6
4*	110	NMLK(GlyGly)VVSR	530.8001	2	−0.24	38.0	3.2e-3
5	164	LLENK(GlyGly)IGVLQNMR	547.9748	3	−0.39	40.4	1.9e-5
6*	185	DNFK(GlyGly)GEENELLLK	831.9213	2	1.20	40.5	2.9e-5
7a*	194	GEENELLLK(GlyGly)SEQEK	880.4403	2	1.40	45.7	5.5e-5
7b*	194	DNFKGEENELLLK(GlyGly)SEQEK	755.3731	3	0.29	47.0	2.6e-4
8	228	LEK(GlyGly)NITR	494.2831	2	0.76	31.5	5.1e-4
9a	244	IQAK(GlyGly)EESEEKEEQVAEFQK	798.3884	3	1.70	48.9	1.1e-7
9b	244	IQAK(GlyGly)EESEEK	652.8205	2	0.67	31.1	3.2e-3
10	250	EESEEK(GlyGly)EEQVAEFQK	651.6294	3	0.86	33.0	1.0e-4
11a*	259	EESEEKEEQVAEFQK(GlyGly)QK	737.0137	3	0.47	32.6	1.6e-4
11b*	259	EEQVAEFQK(GlyGly)QK	739.3688	2	1.50	31.0	5.9e-4
12a	271	EVLLSLFDEK(GlyGly)R	731.8986	2	−0.06	37.8	8.1e-4
12b	271	EVLLSLFDEK(LeuArgGlyGly)R	577.9969	3	0.55	39.5	7.8e-8
13	279	RHEHLLSK(GlyGly)GER	492.5993	3	0.52	25.6	2.7e-3
14a	339	M(Oxidation)LGSK(GlyGly)AGTGGSSGYHYLR	657.9835	3	0.92	57.1	2.5e-8
14b	339	MLGSK(GlyGly)AGTGGSSGYHYLR	978.4751	2	1.90	46.6	1.9e-8
15	380	HWIPK(GlyGly)MNPTIHK	539.2888	3	−0.45	32.2	3.5e-4

*Newly identified in this work.

^#^These two E2–E3 systems were found to be major catalysts in *in vitro* reconstituted hTDO ubiquitination reactions.
