# Supplementary material for: Molecular basis for catalysis and substrate-mediated cellular stabilization of human tryptophan 2,3-dioxygenase
Source: Sci Rep. 2016 Oct 20;6:35169. doi: 10.1038/srep35169 (PMC5071832; doi:10.1038/srep35169)
Supplement: Supplementary Information [file srep35169-s1.pdf]

Molecular basis for catalysis and substrate-mediated cellular stabilization of human tryptophan 2,3-dioxygenase

Ariel Lewis-Ballester,[1,4] Farhad Forouhar,[2,4] Sung-Mi Kim,[3,5] Scott Lew,[2,5]
YongQiang Wang,[3,5] Shay Karkashon,[1,5] Jayaraman Seetharaman,[2]
Dipanwita Batabyal,[1] Bing-Yu Chiang,[1] Munif Hussain,[2]
Maria Almira Correia,[3] Syun-Ru Yeh,[1]* Liang Tong[2]*

[1]Department of Physiology and Biophysics
Albert Einstein College of Medicine
Bronx, NY 10461, USA

[2]Department of Biological Sciences
Northeast Structural Genomics Consortium
Columbia University
New York, NY 10027, USA

[3]Departments of Cellular and Molecular Pharmacology, Pharmaceutical Chemistry, and Bioengineering and Therapeutic Sciences, The Liver Center,
University of California at San Francisco
San Francisco, CA 94158, USA

[4]These authors contributed equally to this work.

[5]These authors contributed equally to this work.

*Co-corresponding authors

Correspondence information

for LT: Phone: (212) 854-5203, E-mail: ltong@columbia.edu

for SRY: Phone: (718) 430-4234, E-mail: syun-ru.yeh@einstein.yu.edu

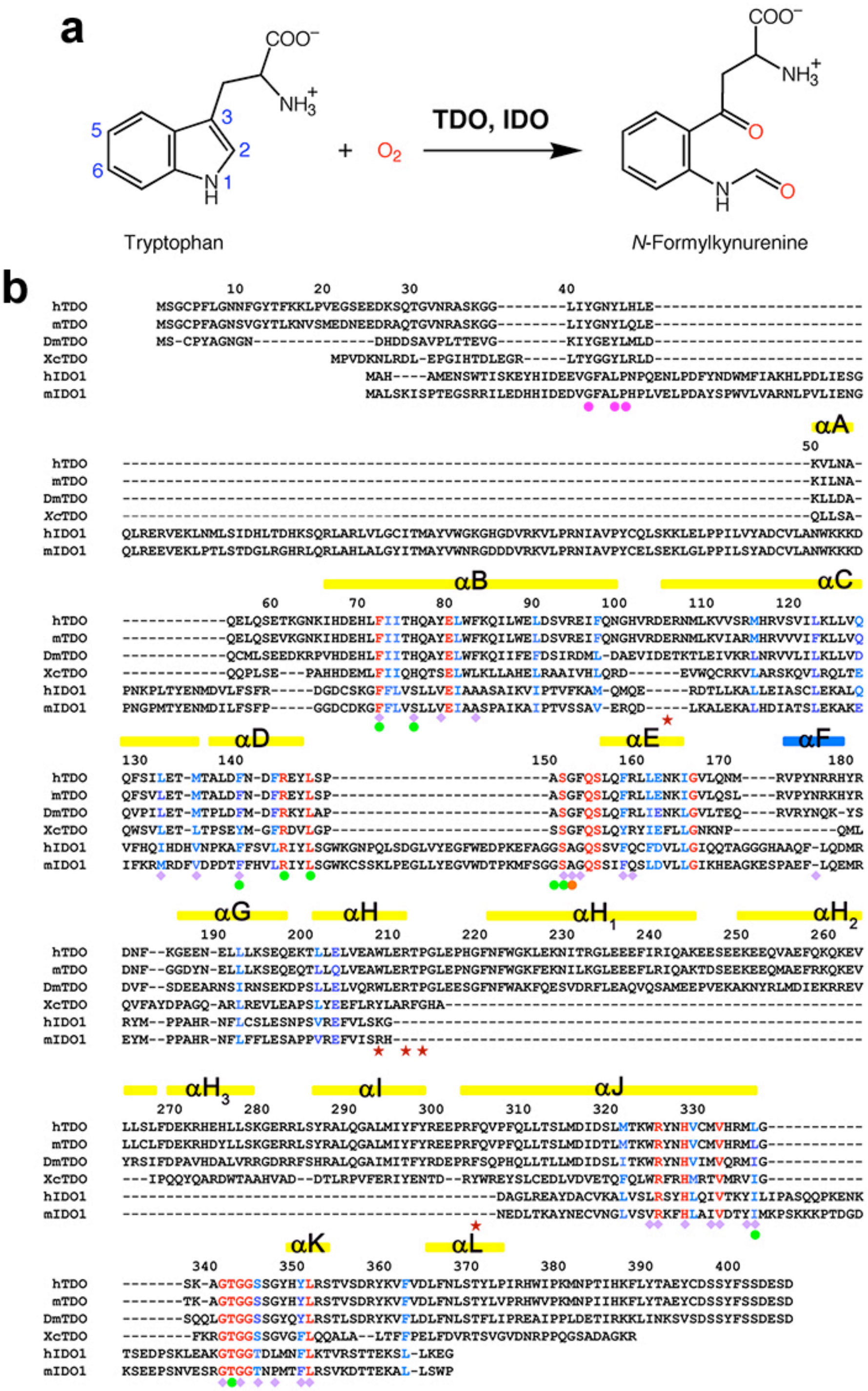

Supplementary Fig. 1. Sequence conservation of TDO and IDO. (**a**). The biochemical reaction catalyzed by TDO and IDO. Both atoms of dioxygen are incorporated into the product. The atom numbering scheme of tryptophan is shown. (**b**). Amino acid sequence alignment of human TDO (hTDO), mouse TDO (mTDO), *Drosophila* TDO (DmTDO), *X. campestris* TDO (XcTDO), human IDO1 (hIDO1) and mouse IDO1 (mIDO1). The purple diamond symbols depict residues involved in binding the heme. Green and orange round symbols denote residues involved in recognition of the substrates *L*-Trp and O_2, respectively. The magenta round symbols indicate conserved residues involved in binding the substrate *L*-Trp from another subunit of the TDO tetramer. The stars indicate residues interacting with the Trp in the exo site. Red: identical residues, blue: similar residues.

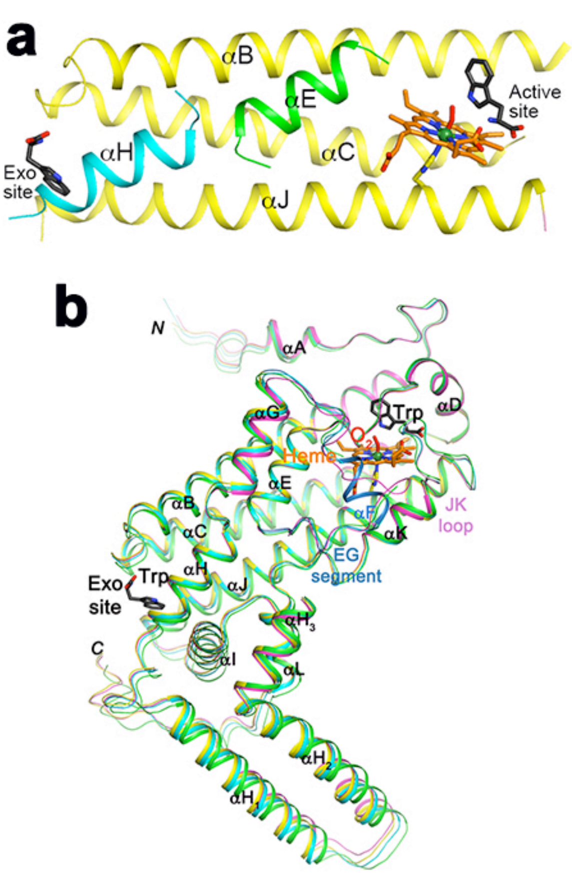

Supplementary Fig. 2. Structure of hTDO. (**a**). Helices αE (green) and αH (cyan) combine to form a long helix, giving rise to a four-helical bundle in hTDO. (**b**). Overlay of the structures of four subunits of hTDO. The subunits are colored as in Fig. 3d. The Trp and O_2 in subunit A are shown as stick models.

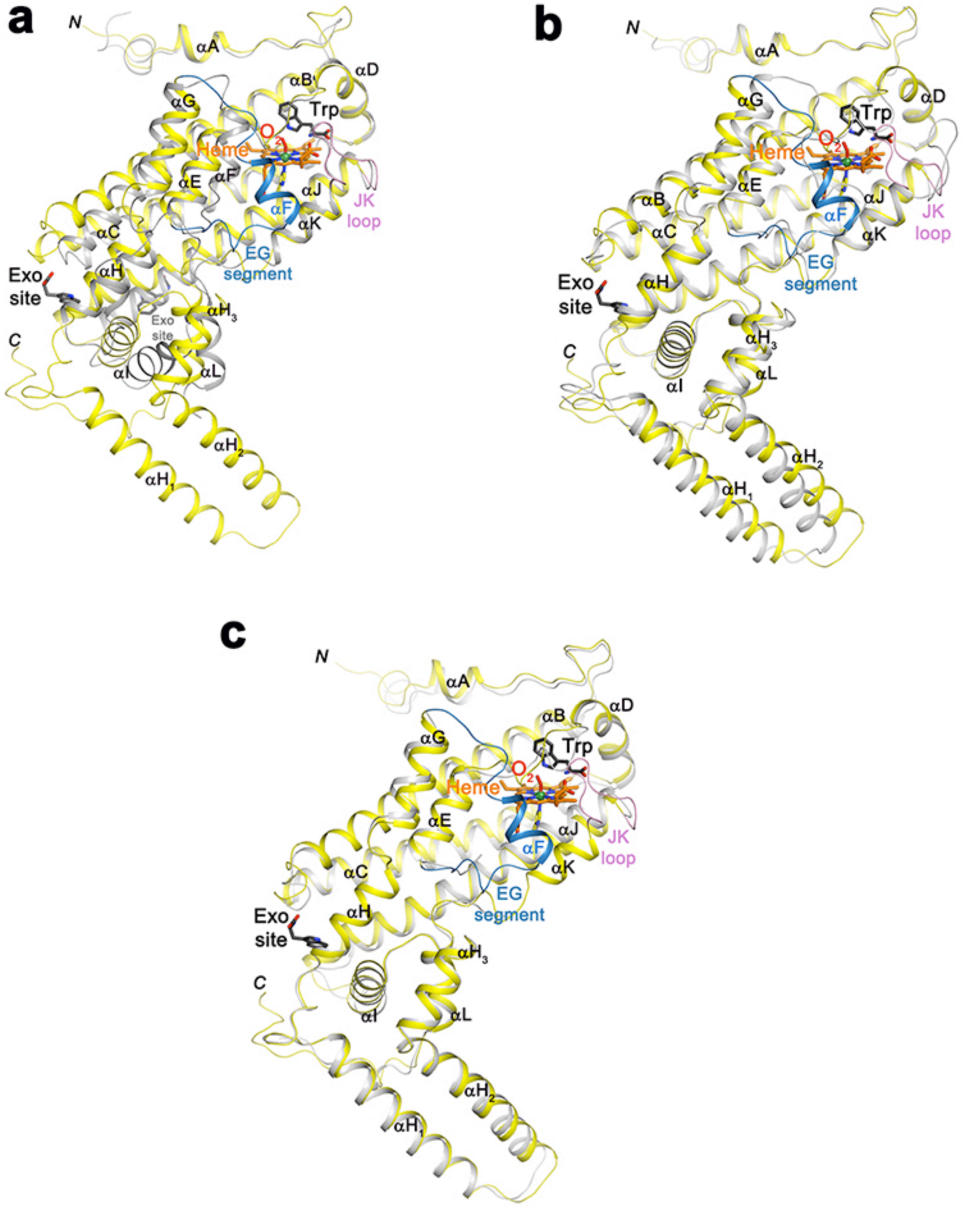

Supplementary Fig. 3. Structural comparison of TDOs. (**a**). Overlay of the structures of hTDO (in color) and XcTDO (in gray) subunits. The rms distance is 1.9 Å for 246 equivalent Cα atoms. Only the heme and *L*-Trp of human TDO are shown, as their bacterial counterparts occupy similar positions. The exo site in XcTDO is labeled in gray. (**b**). Overlay of the structures of hTDO (in color) and DmTDO (in gray) subunits. (**c**). Overlay of the structures of hTDO ternary complex (in color) and apo hTDO (in gray) subunits.

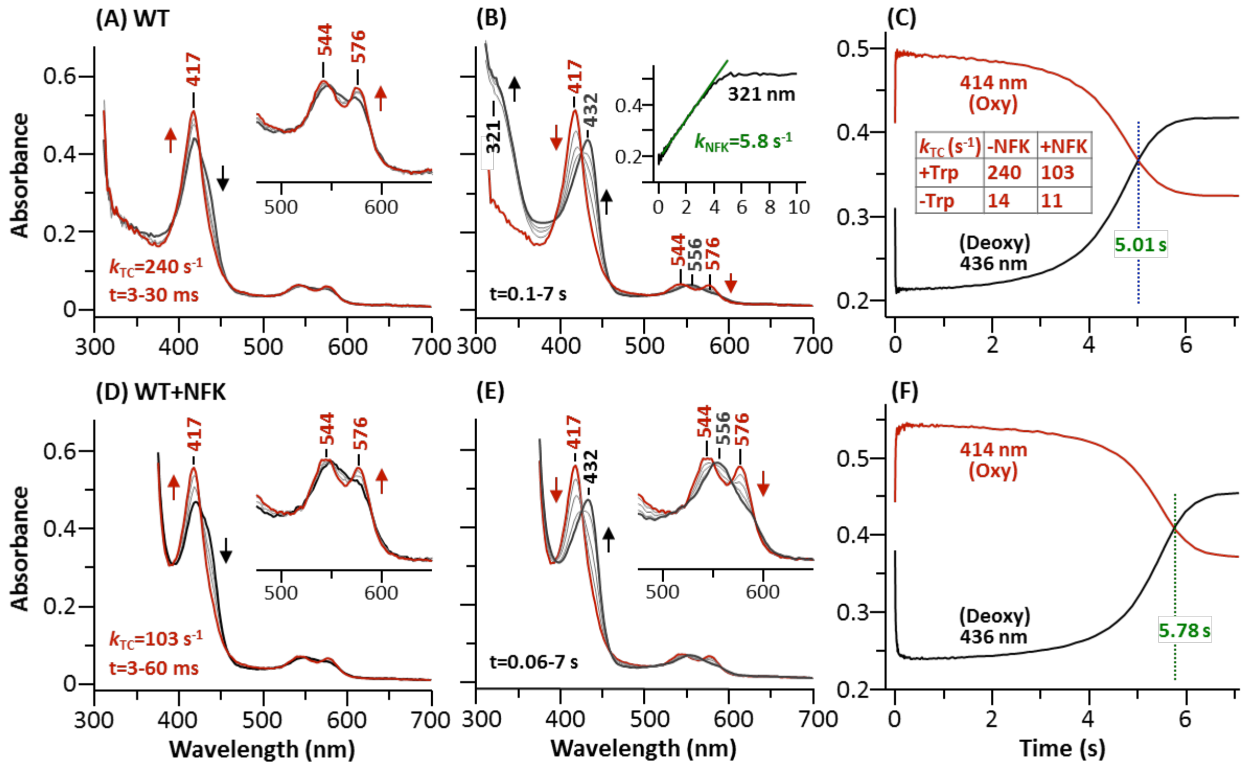

Supplementary Fig. 4. Reduced multiple turnover of the wild-type hTDO reaction by exogenous NFK. (A-C) Spectral kinetics observed following the mixing of deoxy ferrous hTDO with a buffer solution containing 10 mM Trp and 138 μM O_2. The reaction was initiated by Trp and O_2 binding to the deoxy enzyme (λ_{max}=432 nm) to generate the ternary complex (λ_{max}=417 nm), shown as the red trace in (A). As summarized in the table inserted in (C), the rate constant (k_{TC}) was determined to be 240 s^{-1}, which is 17-fold faster than k_{on} (O_2) obtained in the absence of Trp (data not shown), indicating that Trp binding occurred prior to O_2 binding and that pre-binding of Trp to the active site facilitated O_2 binding. The active ternary complex was completely formed at 30 ms, and was almost constantly populated until O_2 in the reaction mixture was consumed, as indicated by the kinetic trace at 414 nm in (C). The constant population of the ternary complex led to linear production of NFK, with a rate of 5.8 s^{-1}, as indicated by the kinetic trace at 321 nm shown in the inset in (B). As the O_2 in the reaction mixture started to be depleted, the ternary complex gradually converted to the deoxy ferrous species with a midpoint of 5.01 s, as indicated by the kinetic traces at 414 and 436 nm in (C); at the same time, NFK production started reaching a plateau. (D-F) Comparable data obtained with the deoxy enzyme pre-incubated with 8 mM NFK. Similar kinetic behavior was observed, but the O_2 consumption rate, as reflected by the oxy→deoxy transition at the end of the reaction is somewhat slower, with a midpoint of 5.78 s instead of 5.01 s, as indicated by the kinetic traces in (F) vs. (C). In addition, as summarized in the table inserted in (C), the apparent k_{TC} was ~2-fold slower. The data suggest that NFK can occupy the active site, thereby impeding Trp/O_2 binding, hence reducing the multiple turnover of the enzyme. It is noted that we were unable to directly determine the product formation rate by monitoring the absorbance at 321 nm, as it was overwhelmed by the excess NFK present in the solution mixture.

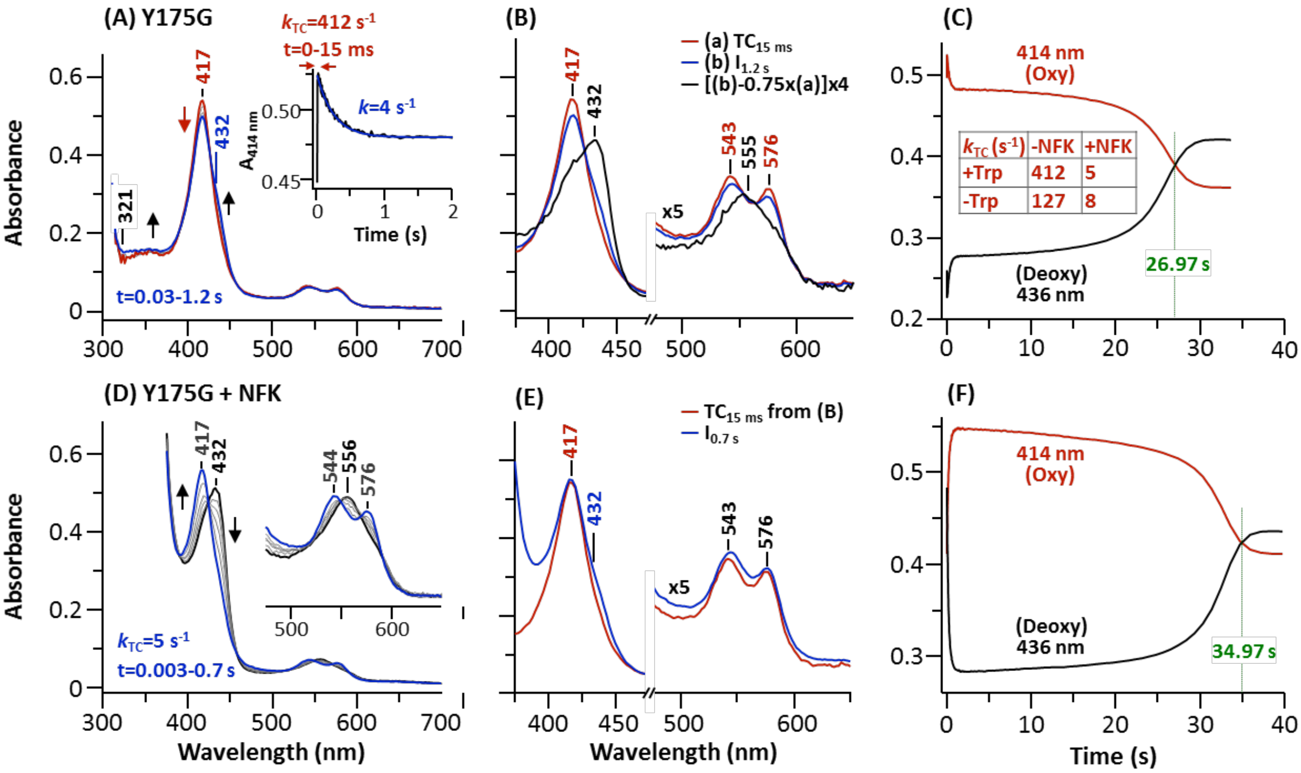

Supplementary Fig. 5. Reduced multiple turnover activity of hTDO by Y175G mutation and exogenous NFK. (A-C) Spectral kinetics observed following the mixing of the deoxy ferrous Y175G mutant with a buffer solution containing 10 mM Trp and 138 μM O_2. The reaction was initiated by the Trp and O_2 binding to the deoxy enzyme (λ_{max}=432 nm) to form the ternary complex (λ_{max}=417 nm), which reached completion at ~15 ms, as highlighted in red in the inset in (A). The rate constant (k_{TC}), 412 s^{-1}, is ~2-fold faster than that of the wild-type reaction, 240 s^{-1}, taken from Supplementary Fig. 5, as summarized in the table inserted in (C). As the ternary complex was fully populated, NFK started to be produced; at the same time, part of the ternary complex started converting back to the deoxy species, as indicated by the increase in the 432 nm shoulder and the concurrent decrease in the 417 nm band shown in (A). The reaction reached completion at 1.2 s, with a rate constant of 4 s^{-1}, as highlighted in blue in the inset in (A). The final spectrum of the transient intermediate species (I), shown as the blue trace in (B), can be deconvoluted into 75% ternary complex (red trace) and 25% deoxy species (black trace), suggesting that one subunit of the tetramer is trapped in an inactive deoxy state. The data imply that once the ternary complex is formed, it can turnover to make NFK, and that the NFK generated from the reaction can bind to the active site of one subunit, thereby arresting it in the deoxy state. The fact that the spectrum of the NFK adduct does not resemble that detected in the in-crystal reaction, shown as the blue spectrum in Fig. 1, indicates that NFK does not directly coordinate to the heme iron. The intermediate I was constantly populated until O_2 in the reaction mixture started to be depleted as indicated by the oxy→deoxy transition, with a midpoint of 26.97 s. (D-F) Comparable data obtained with the deoxy enzyme pre-incubated with 8 mM NFK. The presence of NFK led to 80-fold slower Trp-O_2 binding to form the ternary complex, with k_{TC}=5 s^{-1} instead of 412 s^{-1}, as shown in (D) vs. (A). Similar retardation was observed in the absence of Trp, as summarized in the table inserted in (C). The data support the view that NFK can bind to the active site of the enzyme and that the Trp/O_2 binding reaction is limited by unloading of NFK from the active site. The ternary complex formed at the end of the Trp/O_2 binding reaction, shown as the blue trace in (D) and (E), resembles the intermediate I spectrum shown in (B), indicating that the same intermediate I with one subunit of the tetramer trapped in the inactive deoxy state was formed. The observation that the presence of the exogenous NFK retards O_2 consumption rate, as indicated by the shifts of the midpoint of the oxy→deoxy transition from 26.97 to 34.97 s, shown in (F) vs. (C), supports the scenario that NFK can bind to the active site of the enzyme, thereby retarding its multiple turnover.
